# Higher Frequency of Undetected Acute Coronary Syndrome in Elderly Patients with Chest Pain Who Visited the Emergency Department: A Large-Cohort Retrospective Study

**DOI:** 10.1155/2021/6611051

**Published:** 2021-04-10

**Authors:** Ki Hun Hong, Sung Jin Bae, Dong Hoon Lee, Choung Ah. Lee, Sang Hyun Park, Duk Ho Kim, Eui Chung Kim, Jee Yong Lim, Sangsoo Han, Yoon Hee Choi

**Affiliations:** ^1^Department of Preventive Medicine, College of Medicine, Chung-Ang University, Seoul, Republic of Korea; ^2^Department of Emergency Medicine, College of Medicine, Chung-Ang University, Seoul, Republic of Korea; ^3^Department of Emergency Medicine, Hallym University Dongtan Sacred Heart Hospital, Hwaseong, Republic of Korea; ^4^Department of Emergency Medicine, College of Medicine, The Catholic University of Korea, Seoul, Republic of Korea; ^5^Department of Emergency Medicine, Eulji University, Seoul, Republic of Korea; ^6^Department of Emergency Medicine, CHA Bundang Medical Center, Seongnam, CHA University, Seongnam-si, Gyeonggi-do, Republic of Korea; ^7^Department of Emergency Medicine, Seoul St. Mary's Hospital, Seoul, Republic of Korea; ^8^Department of Emergency Medicine, Soonchunhyang University Bucheon Hospital, Bucheon, Republic of Korea; ^9^Department of Emergency Medicine, Ewha Womans University Mokdong Hospital, College of Medicine, Ewha Womans University, Seoul, Republic of Korea

## Abstract

**Background:**

Acute coronary syndrome (ACS) is a critical disease encountered in the emergency department (ED). Despite the development of diagnostic tools, it may be difficult to diagnose ACS because of atypical symptoms and equivocal test results. We investigated the difference in the rates of revisit and undetected ACS between adult and elderly patients who visited the ED with chest pain.

**Method:**

Data from 11,323 patients who visited the ED with chest pain at university hospitals in Korea were retrospectively analyzed. The cohort was categorized into two age groups: the adult (30–64 years) and elderly (>65 years). Baseline characteristic data (age, sex, vital signs, triage category, etc.) were obtained. We selected patients who revisited the ED within 30 d and investigated whether ACS was diagnosed.

**Result:**

The revisit rate was higher in the elderly (12%) than in the adult group (8.3%). The rate of undetected ACS among the revisited patients was 2.91% (18/7,186) in adults and 6.08% (16/1,998) in elderly patients.

**Conclusion:**

Elderly patients with chest pain had an increased rate of ED revisits and undetected ACS than adult patients. We recommend that old patients should be hospitalized to observe the progression of cardiac complaints or receive short-term follow-up.

## 1. Introduction

Acute coronary syndrome (ACS) is one of the critical diseases encountered in the emergency department (ED). When patients visit the ED with chest pain, emergency physicians (EPs) should assess the chief complaints and include ACS in the differential diagnosis. To improve the accuracy of ACS diagnosis, various diagnostic tools, including an electrocardiogram (ECG) and quantification of biomarkers, are used in the ED. Regarding imaging, echocardiography and coronary computed tomography could help diagnose ACS. However, diagnosis of ACS may be difficult because the imaging findings can be normal, as in the case of unstable angina. Also, some patients present with atypical symptoms and equivocal results, making diagnosis difficult.

Furthermore, ST-segment elevation and increase in troponin levels are definite evidence of ACS. However, in many patients, the result is normal or equivocal, although the patients complain of chest pain. To reduce the risk of misdiagnosis for ACS, EPs observe patients in the ED for several hours and repeat the investigations [1]. Despite observation and rechecking of the ECG and cardiac biomarkers, many patients are discharged from the ED because of negative investigation results. However, some of the discharged patients revisit the ED with the same chief complaint or with other symptoms, and ACS is confirmed in the subsequent evaluation of some of these patients [2]. Moreover, when elderly patients visit the ED for chest pain or similar symptoms, confirmation of ACS diagnosis could be more difficult. Old age constitutes a high-risk factor for ACS, and ACS-associated mortality is high in elderly patients [3]. In addition, ACS patients without chest pain are older than those with chest pain [4], and troponin T has a reduced performance in ACS diagnosis in elderly patients [5]. Moreover, elderly patients tend to receive less aggressive medical and invasive management [6]. Therefore, EPs should be concerned about the possibility of undetected ACS in elderly patients who present with chest pain.

The number of ED revisits by patients diagnosed with ACS undetected in the index visit or an understanding of these proportions according to age has not been elucidated. In this study, we aimed to ascertain the number of patients revisiting the ED and evaluate the proportion of these patients that was subsequently diagnosed with ACS. We investigated the difference in the rates of revisit and undetected ACS between adult and elderly patients who visited the ED with chest pain.

## 2. Materials and Methods

### 2.1. Study Design and Population

This multicentre retrospective cohort study utilized data from the National Emergency Department Information System (NEDIS) that was collected from patients who visited the EDs in eight tertiary university hospitals located in metropolitan areas of South Korea between January 2017 and January 2018. The NEDIS is a nationwide governmental system that has been in operation since 2003 and collects data from more than 150 Korean emergency medical centres. This study was approved by the institutional review board of each hospital, and the need for informed patient consent was waived.

### 2.2. Data Sources and Variables

To analyze ED revisits, we restructured the data in the NEDIS that were collected from eight participating hospitals. Visits that were not for treatment or for unknown reasons were excluded. Moreover, all visits were reported as single visits or revisits, and each revisit was matched to an index visit that occurred just before each revisit. We calculated the frequency and interval of revisits. Frequent ED users (>5 visits within a year) and revisits after a more than 30-day interval were excluded from the study dataset. From this restructured revisit dataset, we applied the following inclusion criteria: chief complaint of “chest pain” and age > 30 years; visits due to injury were excluded. In this study, the chief complaints of “chest pain” included chest pain, chest discomfort, chest palpitation, epigastric pain, epigastric discomfort, and substernal pain.

### 2.3. Outcome Measurement

The primary outcomes were the revisit rate and diagnostic rate of undetected ACS, which were compared between the adult and elderly patients. When patients with chest pain were evaluated according to their index ED visits, some of them were not diagnosed with ACS. Among these non-ACS patients, the revisit rates in adult and elderly patients were analyzed. The secondary outcome was the characteristics of these undetected ACS patients.

### 2.4. Variables

The study variables included mental status, vital signs, the Korean Triage and Acuity Scale (KTAS) score, final diagnosis at discharge, disposition in the ED, and length of ED visit. The KTAS is a Korean triage system that categorizes patients based on a severity scale that ranges from 1 to 5, with 1 being the most severe. The length of ED stay was calculated based on the time of admission and discharge. We categorized the included visits into the adult and elderly groups (30–64 years and >65 years, respectively). Cases of undetected ACS were defined as patients who were not diagnosed with ACS at the index ED visit but were diagnosed with ACS on a revisit to the ED.

After extracting the patients' data from the NEDIS database, we reviewed the electronic medical records (EMR) of patients with undetected ACS to investigate the results of examinations, such as ECG, troponin, and coronary angiography (CAG), and the reason for the revisit.

### 2.5. Statistical Analyses

To assess the differences in age, vital signs, and length of ED stay, we used the independent *t*-test or Mann–Whitney *U*-test for continuous variables. Geometric means and their confidence intervals were calculated to compare differences in the length of ED stay between the study groups. The length of ED stay was nonnormally distributed; therefore, we used their natural log transformation in the analysis. The *t*-test was used to compare the geometric means.

Pearson's chi-square test was used for nominal variables to assess differences in sex, revisit group, mental status, KTAS, diagnosis at hospital, and disposition. Mental status was categorized as either alert or nonalert (i.e., verbal response, painful response, and unresponsiveness) for Pearson's chi-square test or Fisher's exact test. For Pearson's chi-square test, the KTAS score was categorized as levels 1–3 and levels 4 and 5; the diagnosis at the hospital was categorized as ACS and non-ACS; and disposition was categorized as discharge, admission, and death. Continuous variables, such as age and vital signs, are expressed as the mean ± standard deviation, and categorical variables are expressed as numbers and percentages. Significance was set at *p* < 0.05. Statistical analyses were conducted using IBM SPSS statistics version 26.0 (IBM Corp., Armonk, N.Y., USA).

## 3. Results and Discussion

### 3.1. Results

#### 3.1.1. Baseline Characteristics of the Patients

**Figure 1:**
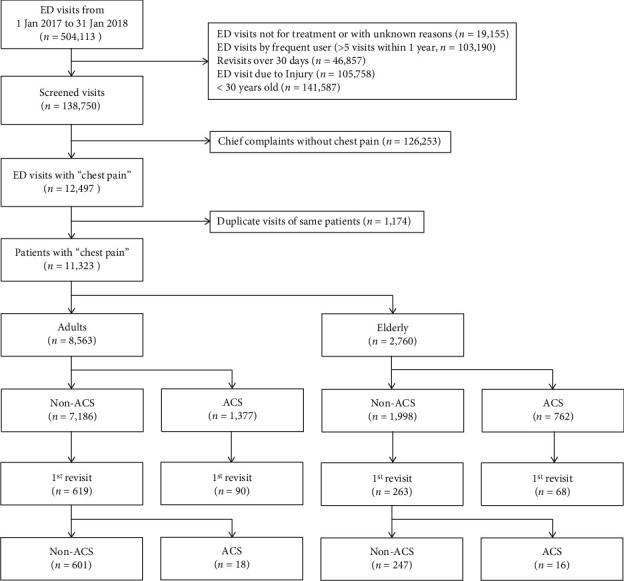
Flow chart of patients enrolled in the study. ED: emergency department; ACS: acute coronary syndrome.

During the study period, 504,113 patients visited the EDs at eight tertiary university hospitals in South Korea; of these, 19,115 patients were excluded because they did not visit the ED for treatment or the reason for their visit was unknown, and 103,190 patients who visited the ED > 5 times within 1 year were excluded. In addition, we excluded 46,857 patients because they revisited the ED > 30 d after the index visit; 198,374 patients were excluded; 105,758 patients were excluded as they visited the ED because of an injury; 141,587 patients who were older than 30 years were excluded; and 126,253 patients who did not have chest pain as the chief complaint were excluded. Finally, 11,323 patients were enrolled in this study (Figure 1). Among the 8,563 patients who were in the age group of 30–64 years and had a complaint of chest pain on the index ED visit, 18 patients were not diagnosed with ACS on their index ED visit, but ACS was confirmed on ED revisit within 30 d from the index visit. In 2,760 elderly patients (>65 years), undetected ACS was observed in 16 patients (Figure 1).

The mean age of patients with chest pain was 46.9 ± 10.0 and 74.3 ± 6.8 years in the adult and elderly groups, respectively (Table 1). Chest pain was common in males in the adult group, whereas it was more common in females in the elderly group (51.4% and 54.1% in the adult and elderly groups, respectively, *p* < 0.001). The revisit rate was higher in the elderly than in the adult group (12.0% and 8.3%, respectively, *p* < 0.001). A triage level of >3 in the KTAS was more common in the elderly than in adults (86.7% and 75.9%, respectively, *p* < 0.001). The rate of admission was 44% and 22% in the elderly and adult groups, respectively (*p* < 0.001). In elderly patients, the mean length of ED stay was 194.0 minutes, which was longer than the 150.2 minutes recorded for the adult group (*p* < 0.001).

#### 3.1.2. Revisit Rate and Diagnostic Rate of Undetected ACS in the Adult and Elderly Patient Groups

When patients with chest pain were evaluated according to their index ED visits, 7,186 of 8,563 adult patients and 1,998 of 2,760 elderly patients were not diagnosed with ACS (Figure 1). Among these non-ACS patients, the revisit rate was 8.61% (619/7,186) in adults and 13.16% (263/1,998) in elderly patients (*p* < 0.001, Table 2). The diagnostic rate of undetected ACS among patients who revisited the ED within 30 d was 2.91% (18/7,186) and 6.08% (16/1,998) in the adult and elderly patient groups, respectively. There was a significant difference in the diagnostic rate of undetected ACS between the adult and elderly patients (*p* = 0.025).

#### 3.1.3. Characteristics of Patients with Undetected ACS on the Index ED Visit

There were 18 and 16 patients in the adult and elderly patient groups, respectively, who were diagnosed with ACS during the revisit and not at the time of the index visit. Among patients with undetected ACS, a higher proportion of female patients was noted in the adult group than in the elderly group (83.3% and 43.7%, respectively, *p* < 0.001, Table 3). The intergroup differences in vital signs, triage levels, and disposition were not statistically significant. The length of ED stay was longer in the elderly than in adult patients (324.3 and 166.5 minutes, respectively, *p* < 0.001).

#### 3.1.4. Diagnostic Evaluation Results of Patients with Undetected ACS

Table 4 shows the diagnostic results of patients with undetected ACS at their index visit and on revisit. They include ECG, cardiac enzyme, and CAG results. At the index visit, there were no specific ECG findings, such as ST elevation or depression, in the adult patients. In the elderly, 1 of 16 patients had ST depression in the ECG. On their revisit, ECG change was found in 4 of 18 adult patients and 2 of 16 elderly patients. Besides, 4 adults and 2 elderly patients had normal cardiac enzyme results at the first visit but were elevated on the revisit.

## 4. Discussion

This is the first study to investigate the characteristics of patients with chest pain complaints who revisited the ED in South Korea. This study compared the rate of ED revisit between the elderly and adult patients and the proportion of patients diagnosed with ACS based on revisits. We found that older patients need a more cautious diagnosis when they visit the ED with chest pain complaints. We showed that 9.6% of all patients who complained of chest pain revisited the ED within 30 days, and the rate of revisit of elderly patients was higher at 13.2% compared to adult patients at 8.6%. The diagnostic rates of ACS among patients with chest pain at the index visit were 16.0% and 27.6% in adult and elderly patients, respectively, while the corresponding rates at revisit were 2.91% and 6.0% (Figure 1). The revisit rate in patients with chest pain and the rate of undetected ACS at the index ED visit were higher in elderly patients (age > 65 years) than in adult patients (Table 2).

International cardiac guidelines recommend that patients who visit the ED with chest pain complaints should be assessed with a risk stratification tool, and over the years, a number of protocols have been developed [7]. Risk-scoring tools, such as the GRACE score, the HEART score, and the TIMI score, are widely applied [8–10]. While these risk-scoring tools use different scoring variables, all of them include age as old age is a risk factor for ACS. Therefore, we performed an age-stratified analysis of the characteristics of patients who revisited the ED. EPs do not want their patients to revisit the facility with the same chief complaints. When patients with chest pain revisit the ED within a short interval, they should be evaluated for ACS.

To diagnose ACS, various diagnostic tools, such as echocardiography, ECG, and biomarkers, are used. Cardiac troponin is widely used and one of the most popular and effective biomarkers for ACS. According to the 2015 European Society of Cardiology guidelines, the measurement of a cardiac biomarker, especially high-sensitivity troponin, had high sensitivity and specificity; therefore, it was to be mandatorily ascertained in all patients suspected to have ACS [11]. Troponin levels in peripheral blood increase slowly; thus, clinical practice guidelines recommend a second measurement of troponin over specific time intervals to exclude ACS [12]. In each algorithm, Reichlin and colleagues and the United Kingdom National Institute for Health and Care Excellence (NICE) have included the measurement of cardiac troponin at 2 h intervals to rule out ACS [13]. Other studies have suggested that the interval should be 3 h, and many studies were conducted on the intervals at which troponin was measured to increase its sensitivity and specificity in ACS diagnosis [12, 14]. As a result, patients who visit the hospital complaining of chest pain would stay longer in the emergency room. In this study, the ED length of stay was longer for the same reason, such as repeated tests. Characteristically, despite the fact that elderly patients stayed longer in the ED than adults and received a careful examination, they had a higher rate of revisits than adult patients. Even in patients with undetected ACS, the elderly stayed in the ED for nearly twice as long as adults during the index visit and underwent more and repeated tests. Advances in laboratory technology have made it possible to measure very low concentrations of troponin, thereby increasing the sensitivity of troponin detection. However, cardiac troponin has reduced ACS diagnostic performance in the elderly [5]. In this study, troponin T levels of patients diagnosed with undetected ACS were elevated in only 1 of 18 patients at the time of the first ED visit and were clearly elevated in four patients at the time of ED revisit and being diagnosed with ACS among the adult patients. In the elderly group, only 1 of 16 patients had elevated troponin T levels at the time of the first visit, and the levels in three patients were clearly elevated when ACS was diagnosed after the revisit (Table 4). Even if troponin T levels are normal, further reexamination is needed within a short period, especially in older patients.

Previous studies on the diagnostic rate of ACS have varied results. Hoorweg et al. [15] reported that 8.4% of patients with chest pain were diagnosed with life-threatening diseases such as acute myocardial infarction. In another study, 72.4% of patients with chest pain lacked a specific underlying cause, and most of them were not diagnosed with any type of cardiovascular disease over the subsequent 6 months [16]. Additionally, there are several studies on the ED revisit, but they have varied findings because of different inclusion criteria, the interval of revisit, and primary outcomes [17]. In 1993, Kelly et al. reported that unscheduled revisits accounted for 2% of patient encounters [18]. A 2020 study in Taiwan reported that the overall revisit rate was 5.47% within 72 h [19]. In a study conducted in 2015 in the USA, the adult revisit rate within 3 days was 8.2% [20]. In this study, the interval for revisit was set as 30 days. Using a similar definition, Biese et al. [21] reported that the 30-day return rate in elderly patients varied from 18% to 39%. This study's result is similar to that of a previous study, which evaluated the overall prevalence of ACS diagnosed within 30 days after ED visit and was conducted in Texas, USA [2]. Moreover, the high rate of revisits in the elderly patients was consistent with the rate reported in a previous study which revealed that it was more challenging to diagnose ACS in elderly patients [3].

This study analyzed data from a large number of patients who visited the ED; however, there are several limitations. This was a retrospective study of patients who visited the ED with complaints of chest pain. Thus, selection bias may be present. This study excluded the visits for unknown reasons, which were 179 of the total 504,113 ED visits, and this could be a source of bias. We studied data from the NEDIS database; therefore, we had no access to information on the patient's medical history and other signs, such as diaphoresis, dyspnea, or radiating pain, that are suggestive of ACS. However, we do not believe that these signs at the time of index visit influenced discharge, admission, or revisit. Thus, it is unlikely that the signs and symptoms at the index visit would have confounded our results. We excluded frequent users who repeatedly visited the ED > 5 times a year (Figure 1). There is a possibility that some of these patients have been diagnosed with ACS and then received treatment. Furthermore, we did not include patients who revisited other hospitals due to various reasons, such as their home base. However, we do not expect the number of these patients to be large.

We found an increased risk of ED revisits in elderly patients with chest pain compared to adult patients. Moreover, elderly patients had a higher rate of undetected ACS than adults. Thus, even if symptoms improve during the first visit and there are no detectable abnormalities on the ECG or cardiac marker levels, we recommend that elderly patients should be hospitalized to monitor their progress or receive short-term follow-up in the Department of Cardiology.

## Figures and Tables

**Table 1:** General characteristics of patients with chest pain at the index visit.

	Adult (*n* = 8,563)	Elderly (*n* = 2,760)	*p* value
Age (years)	46.9 ± 10.0	74.3 ± 6.8	<0.001
Sex			<0.001
Male	4,403 (51.4)	1,266 (45.9)	
Female	4,160 (48.6)	1,494 (54.1)	
Revisit group			<0.001
Single visit	7,854 (91.7)	2,429 (88.0)	
Revisit	709 (8.3)	331 (12.0)	
Mental status			0.002
Alert	8,538 (99.7)	2,740 (99.3)	
Verbal response	22 (0.3)	14 (0.5)	
Painful response	2 (0.0)	5 (0.2)	
Unresponsive	1 (0.0)	1 (0.0)	
Vital sign			
Mean ABP	108.4 ± 17.5	109.0 ± 19.6	0.132
Pulse rate	82.7 ± 16.9	81.9 ± 19.9	0.029
Respiratory rate	19.5 ± 2.9	19.6 ± 2.6	0.244
Body temperature (°C)	36.6 ± 1.1	36.6 ± 1.5	0.042
KTAS			<0.001
Level 1	14 (0.2)	19 (0.7)	
Level 2	2,621 (30.6)	1,373 (49.8)	
Level 3	3,866 (45.1)	1,000 (36.2)	
Level 4	1,945 (22.7)	350 (12.7)	
Level 5	117 (1.4)	18 (0.6)	
Diagnosis			<0.001
Acute coronary syndrome	1,376 (16.1)	762 (27.6)	
Non-ACS	7,187 (83.9)	1,998 (72.4)	
Acute gastritis	1,720 (20.1)	246 (8.9)	
Chest pain, unspecified	1,705 (19.9)	548 (19.9)	
Others	3,762 (43.9)	1,204 (43.6)	
Disposition			<0.001
Discharged	6,664 (77.8)	1,520 (55.1)	
Symptoms improved	6,205 (72.5)	1,370 (49.6)	
Against medical advice	440 (5.1)	145 (5.3)	
Other reasons	19 (0.2)	5 (0.2)	
Admission	1,897 (22.2)	1,228 (44.4)	
General ward	1,162 (13.6)	658 (23.8)	
Intensive care unit	667 (7.8)	517 (18.7)	
Transfer	67 (0.8)	53 (1.9)	
Death	2 (0.0)	12 (0.4)	
Length of ED stay (min)^∗^	150.2 (147.8–152.6)	194.0 (187.6–200.6)	<0.001

Values are expressed as number (%), mean ± standard deviation, or median (range) as appropriate. ^∗^Geometric mean with 95% confidence interval. KTAS: Korean Triage and Acuity Scale; MI: myocardial infarction; ACS: acute coronary syndrome; ED: emergency department.

**Table 2:** Differences in the rates of revisiting and undetected ACS between the adult and elderly patient groups.

Characteristics	Adult (*n* = 7,186)	Elderly (*n* = 1,998)	*p* value
Revisit within 30 d			<0.001
Single visit	6,567 (91.4)	1,735 (86.8)	
Multiple revisit	619 (8.6)	263 (13.2)	
Diagnosis of ACS at ED revisit			0.025
ACS	18 (2.9)	16 (6.1)	
Not ACS	601 (97.1)	247 (93.9)	

Values are expressed as number (%). ACS: acute coronary syndrome.

**Table 3:** Comparison of the characteristics of patients with undetected acute coronary syndrome at the index visit.

	Adult (*n* = 18)	Elderly (*n* = 16)	*p* value
Age (years)	52.8 ± 7.8	75.4 ± 7.1	<0.001
Sex			<0.001
Male	15 (83.3)	7 (43.7)	
Female	3 (16.7)	9 (56.3)	
Mental status			
Alert	18 (100.0)	16 (100.0)	
Vital sign			
Mean ABP	109.9 ± 13.4	109.5 ± 16.9	0.942
Pulse rate	72.6 ± 17.7	81.8 ± 19.5	0.157
Respiratory rate	19.3 ± 1.5	19.3 ± 1.7	0.970
Body temperature (°C)	36.4 ± 0.3	36.7 ± 0.4	0.035
KTAS			0.347
Level 2	10 (55.6)	8 (50.0)	
Level 3	5 (27.8)	7 (43.7)	
Level 4	3 (16.7)	0 (0.0)	
Level 5	0 (0.0)	1 (6.3)	
Diagnosis			0.874
Not specific, diagnosed	16 (83.3)	13 (81.2)	
Chest pain, unspecified	9 (50.0)	9 (56.2)	
Others	6 (33.3)	4 (25.0)	
Gastrointestinal diseases	3 (16.7)	3 (18.8)	
Acute gastritis	3 (16.7)	2 (12.5)	
Gastroenteritis	0 (0.0)	1 (6.3)	
Disposition			0.282
Discharged	18 (100.0)	15 (93.7)	
Symptoms improved	14 (77.8)	13 (81.2)	
Against medical advice	4 (22.2)	2 (12.5)	
Admission	0 (0.0)	1 (6.3)	
General ward	0 (0.0)	1 (6.3)	
Length of ED stay (min)^∗^	166.5 (129.3–214.3)	324.3 (203.0–518.2)	<0.001

Values are expressed as number (%), mean ± standard deviation, or median (range) as appropriate. ^∗^Geometric mean with 95% confidence interval. KTAS: Korean Triage and Acuity Scale; ED: emergency department.

**Table 4:** Summary of diagnostic evaluation results of patients with undetected ACS.

Reasons for revisit	Age/sex	Index visit		Revisit		CAG
		ECG	Cardiac enzyme	ECG	Cardiac enzyme	
Adults						
Discharged against medical advice (*n* = 4)	42/M	Normal	Elevated	Nonspecific ST change	Borderline	mLAD: 40% stenosis
	47/M	Nonspecific ST change	Borderline	Nonspecific ST change	Elevated	pLCX: chronic total occlusion, dRCA: thrombotic occlusion
	62/M	Normal	Normal	Normal	Normal	mRCA: 90% stenosis
	50/M	Normal	Normal	Unchecked	Unchecked	Unchecked

Recurrent symptom (*n* = 13)	42/M	Normal	Normal	ST elevation	Elevated	mLAD: spasm
	61/M	RBBB	Normal	ST depression	Elevated	LAD: 90% stenosis
	52/M	Normal	Normal	Normal	Elevated	pLCX: 70-80% stenosis
	45/M	Normal	Normal	Normal	Normal	mLAD and mRCA: 20% stenosis
	57/M	Normal	Normal	Normal	Normal	mLAD: total occlusion
	61/M	Normal	Normal	Normal	Normal	p-dLCX: 80% stenosis, mLAD: 60% stenosis
	63/M	Normal	Normal	Normal	Normal	pLAD and mLCX: spasm
	39; 53; 54/F, 58; 63/M	Normal	Normal	Normal	Normal	Unchecked
	54/M	Normal	Unchecked	Normal	Normal	Normal

Different symptom (*n* = 1)	47/M	Normal	Normal	Normal	Normal	Normal

Elderly						
Discharged against medical advice (*n* = 2)	76/F	Nonspecific ST change	Elevated	Nonspecific ST change	Elevated	mLAD: 70% stenosis, pLCX: 40% stenosis, pRCA total occlusion
	77/M	Normal	Normal	Unchecked	Unchecked	mLAD: 90% stenosis

Suboptimal management (*n* = 4)	70/F	Normal	Borderline	Normal	Normal	Normal
	76/M	Normal	Borderline	ST depression	Borderline	pRCA: 80% stenosis
	82/F	Normal	Borderline	T inversion	Borderline	Unchecked
	84/F	RBBB	Borderline	RBBB	Borderline	Unchecked

Recurrent symptom (*n* = 9)	67/F^∗^	ST depression	Normal	ST depression	Elevated	pLAD: chronic total occlusion, p-dLCX: 90% stenosis, mRCA: 90% stenosis, dRCA: 80% stenosis
	70/M	Normal	Normal	Nonspecific ST change	Normal	mLAD: 95% stenosis
	76/F	Normal	Normal	Nonspecific ST change	Normal	mLAD: 50% stenosis
	82/M	Normal	Normal	Nonspecific ST change	Normal	dLAD: 50% stenosis, LCX: 40% stenosis, pRCA: 35% stenosis
	65; 80/M, 85/F	Normal	Normal	Normal	Normal	mLCX: 40% stenosis
	80/M	Normal	Normal	Normal	Normal	mLAD and mRCA: 20% stenosis
	85/F	Normal	Normal	Normal	Normal	pLAD: 70% stenosis
	66/M, 67/F^∗∗^	Normal	Normal	Normal	Normal	Unchecked

Different symptom (*n* = 1)	84/F	Normal	Normal	Nonspecific ST change	Elevated	Unchecked

^∗^Death in intensive care unit; ^∗∗^general ward admission at index visit. ACS: acute coronary syndrome; CAG: coronary angiography; ECG: electrocardiogram; RBBB: right bundle branch block; LAD: left anterior descending coronary artery; LCX: left circumflex coronary artery; RCA: right coronary artery; m: middle; p: proximal; d: distal.

## Data Availability

The datasets used and analyzed during the current study are available from the corresponding author on reasonable request.
